# Supplementary material for: Interference and co-existence of staphylococci and Cutibacterium acnes within the healthy human skin microbiome
Source: Commun Biol. 2022 Sep 7;5:923. doi: 10.1038/s42003-022-03897-6 (PMC9452508; doi:10.1038/s42003-022-03897-6)
Supplement: Supplementary file 2 — Supplementary Information [file 42003_2022_3897_MOESM2_ESM.pdf]

Supplementary information: supplementary figures 1-5 and supplementary tables 1-4

Interference and co-existence of staphylococci and *Cutibacterium acnes* within the healthy human skin microbiome

Charlotte Marie Ahle[1,2,*], Kristian Stødkilde-Jørgensen[3], Anja Poehlein[4], Mechthild Bömeke[4], Wolfgang R. Streit[2], Horst Wenck[1], Jörn Hendrik Reuter[1], Jennifer Hüpeden[1], Holger Brüggemann[3,*]

[1]Beiersdorf AG, Research & Development, Front End Innovation, 20245 Hamburg, Germany
[2]Department of Microbiology and Biotechnology, University of Hamburg, 22609 Hamburg, Germany
[3]Department of Biomedicine, Aarhus University, 8000 Aarhus, Denmark
[4]Department of Genomic and Applied Microbiology, Institute of Microbiology and Genetics, University of Göttingen, 37073 Göttingen, Germany

* corresponding authors:
Charlotte Marie Ahle: Charlotte.Marie.Ahle@studium.uni-hamburg.de
Holger Brüggemann: brueggemann@biomed.au.dk

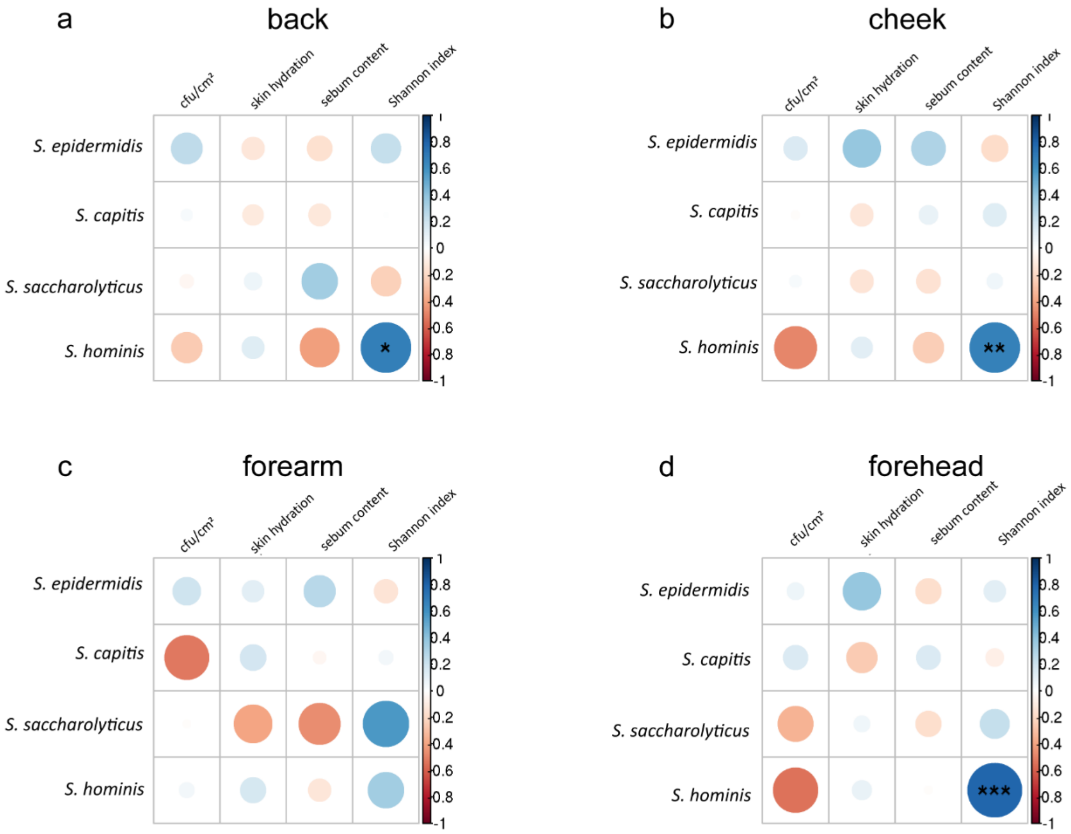

Supplementary Figure 1 Spearman correlation between staphylococcal species abundances (determined by amplicon-based NGS) and skin parameters. This analysis was performed for each skin site separately: **a** back **b** cheek **c** forearm **d** forehead (FDR-adjusted p-value, $*p \leq 0.05$, $**p \leq 0.01$, $***p \leq 0.001$).

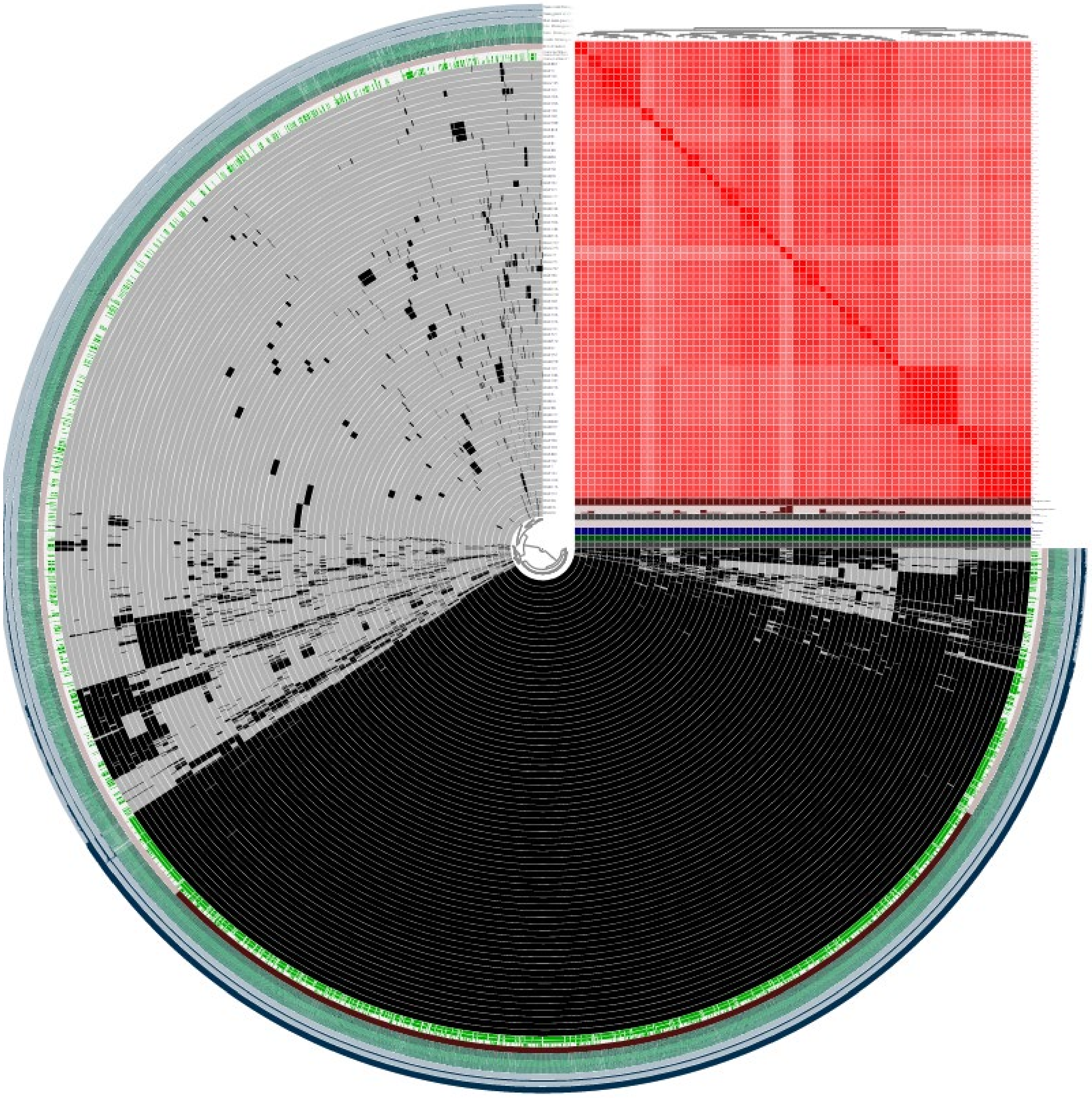

Supplementary Figure 2 Pan-genome of 69 *S. epidermidis* strains isolated in this study.
Pan-genome analysis of the 69 strains was done with ANVIO. The pan-genome is composed of the core genome (i.e. genes shared by all strains) and a large accessory genomes (i.e. genes specific to single strains or subset of strains). Presence (black) and absence (grey) of gene clusters are depicted. The strains are sorted according to their average nucleotide identity (ANI) (red square; a higher ANI is depicted by a darker red color).

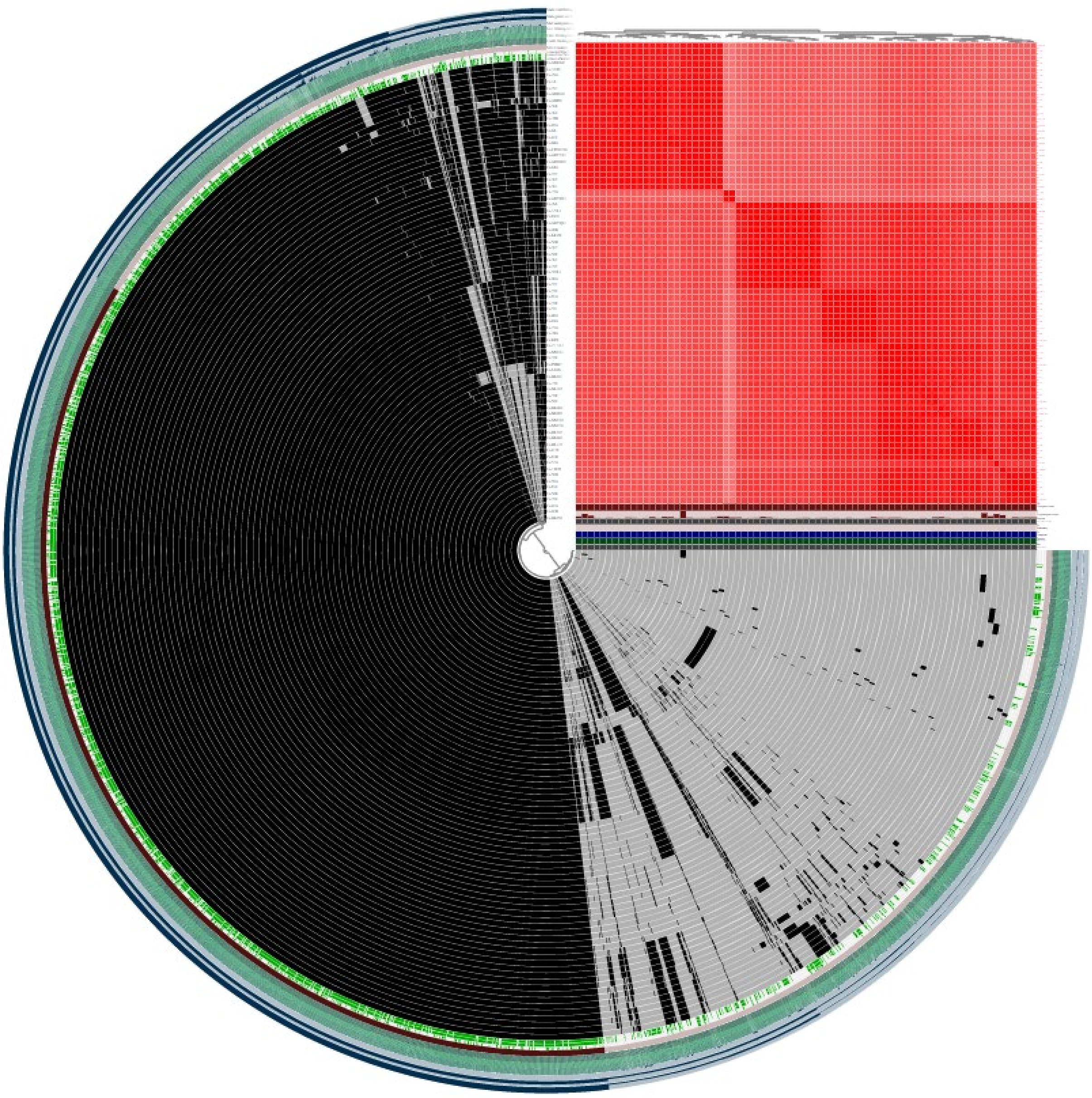

Supplementary Figure 3 Pan-genome of 75 *C. acnes* strains covering all SLST classes.

Pan-genome analysis of the 75 strains was done with ANVIO. The 75 strains were chosen among all published *C. acnes* genomes and included strains from all 10 SLST classes (Supplementary data 7). The pan-genome is composed of the core genome (i.e. genes shared by all strains) and a large accessory genomes (i.e. genes specific to single strains or subset of strains). Presence (black) and absence (grey) of gene clusters are depicted. The strains are sorted according to their average nucleotide identity (ANI) (red square; a higher ANI is depicted by darker red).

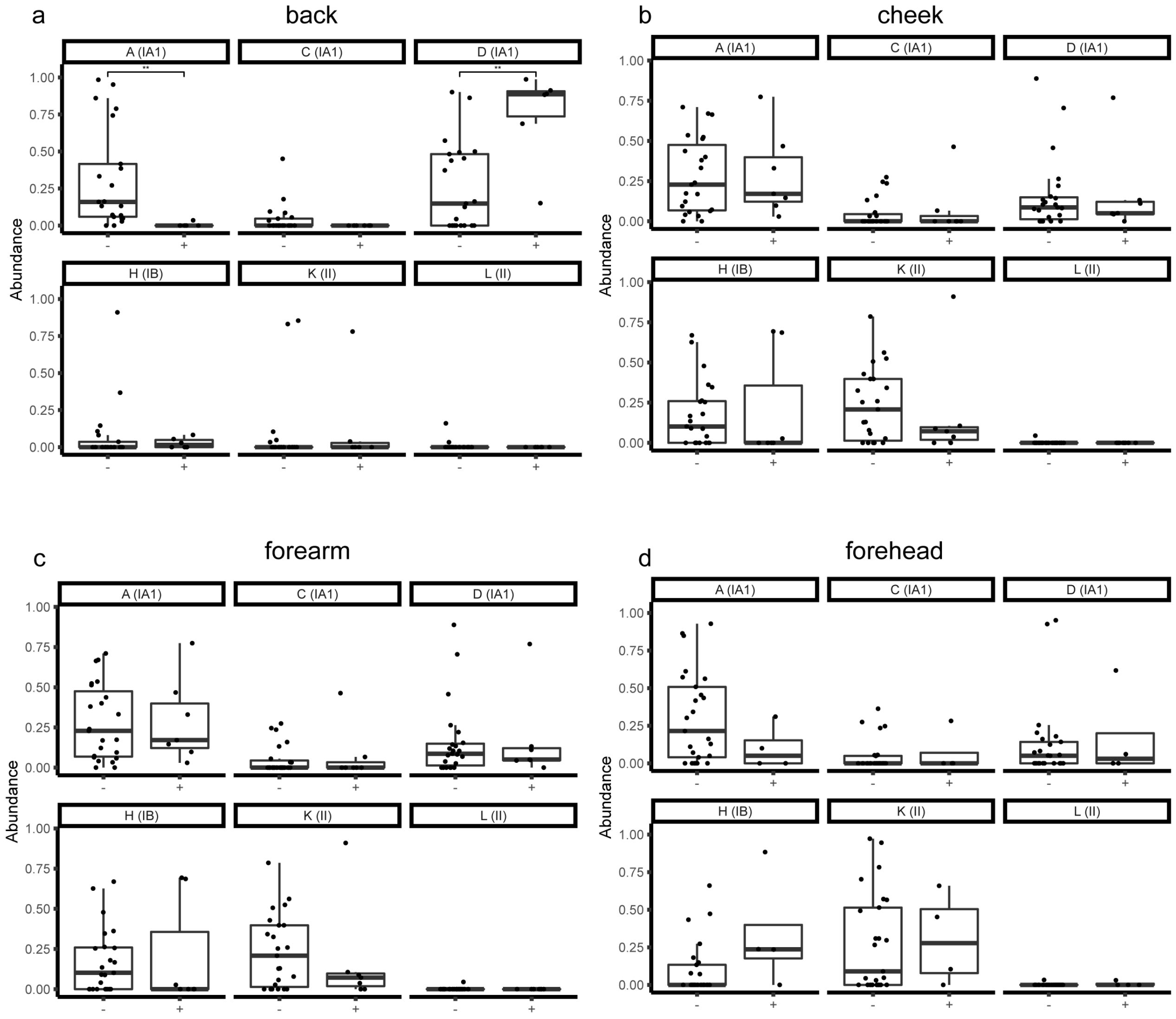

Supplementary Figure 4 Staphylococcal strains with antimicrobial activity influence *C. acnes* populations. Depicted are relative abundances of six *C. acnes* SLST classes (A, C, D, H, K, L) on **a** back **b** cheek **c** forearm **d** forehead skin sites with (+) or without (-) staphylococcal strains with antimicrobial activity (back skin samples: n=21 (-), n=6 (+); cheek skin samples: n=23 (-), n=7 (+); forearm skin samples: n=23 (-), n=4 (+); forehead skin samples: n=25 (-), n=4 (+). FDR-adjusted p-value, **$p \leq 0.01$. Unpaired Wilcoxon test). Middle lines of boxplots indicate the median. Lower and upper lines represent the first and third quartiles. Whiskers show the 1.5x inter-quartile ranges.

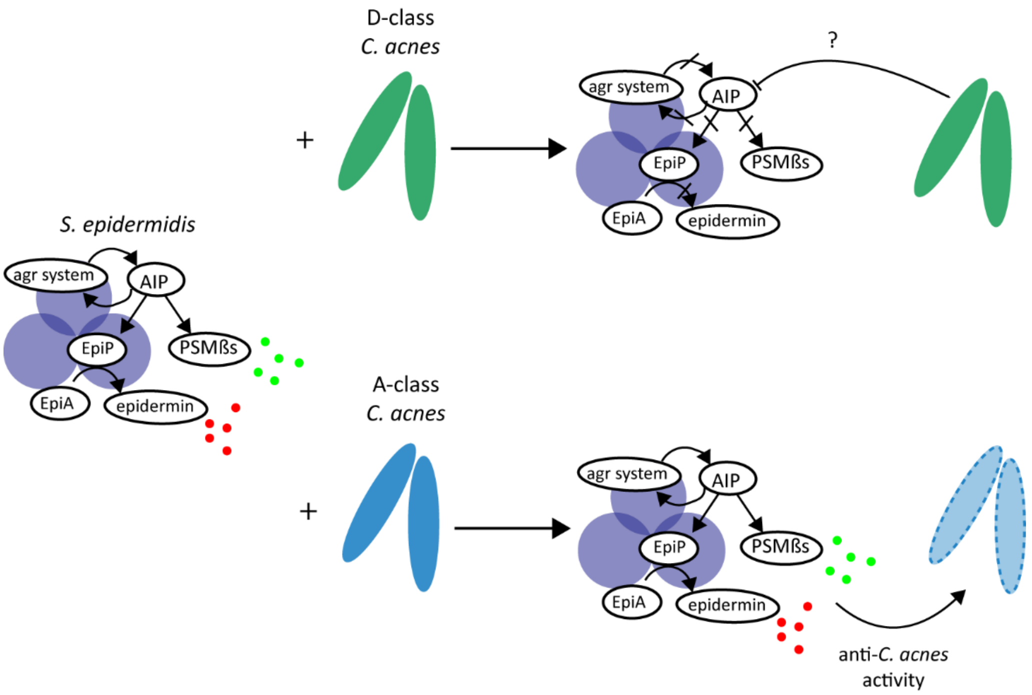

Supplementary Figure 5 Model of interference of antimicrobially active *S. epidermidis* with sensitive and tolerant strains of *C. acnes*, respectively.
S. epidermidis with anti-*C. acnes* activity produces epidermin and phenol-soluble modulins (i.e. PSMßs) that are the main factors responsible for *C. acnes* killing. Genes coding for PSMßs and EpiP, a protease important for the conversion of the peptide precursor EpiA in the active epidermin, are under the control of the *agr* quorum sensing system. Tolerant D-class *C. acnes* inhibits the *agr* system by a so far unknown mechanism, that might involve the direct or indirect inhibition of autoinducing peptide (AIP) production; thus, both epidermin and PSMßs are not secreted in the presence of tolerant D- class *C. acnes*. In contrast, sensitive A-class *C. acnes* does not have any impact on the *agr* system of *S. epidermidis* and is therefore killed by epidermin and PSMßs.

Supplementary Table 1 ANCOM-BC results (coefficient and adjusted p-value) for differences in abundances of staphylococcal species between skin sites (back = Ba, Ch = Cheek, Fa = Forearm, Fh = Forehead)

	Ba - Ch		Ba - Fa		Ba - Fh		Ch - Fa		Ch - Fh		Fa - Fh	
	coeff.	adj. p	coeff.	adj. p	coeff.	adj. p	coeff.	adj. p	coeff.	adj. p	coeff.	adj. p
S. epidermidis	0.13	0.35	0.06	1.00	0.13	0.45	-0.12	0.68	-0.01	1.00	0.07	1.00
S. capitis	0.01	1.00	0.00	1.00	0.01	1.00	-0.06	1.00	-0.02	1.00	-0.01	1.00
S. saccharolyticus	-0.10	0.42	-0.03	1.00	-0.06	1.00	0.03	1.00	0.04	1.00	-0.04	1.00
S. hominis	-0.05	0.63	0.18	0.00	-0.02	1.00	0.19	0.03	0.01	1.00	-0.22	0.00
below threshold	0.00	1.00	0.10	0.00	0.00	1.00	0.06	0.68	-0.01	1.00	-0.11	0.02

Supplementary Table 2 ANCOM-BC results (coefficient and adjusted p-value) for differences in abundances of *C. acnes* SLST classes (A, C, D, E, F, H, G, K, L) between skin sites (back = Ba, Ch = Cheek, Fa = Forearm, Fh = Forehead)

	Ba - Ch		Ba - Fa		Ba - Fh		Ch - Fa		Ch - Fh		Fa - Fh	
	coeff.	adj. p	coeff.	adj. p	coeff.	adj. p	coeff.	adj. p	coeff.	adj. p	coeff.	adj. p
A (IA1)	0.04	1.00	0.04	1.00	0.03	1.00	0.00	1.00	-0.01	1.00	-0.01	1.00
C (IA1)	0.02	1.00	0.03	1.00	0.02	1.00	0.01	1.00	-0.01	1.00	-0.02	1.00
D (IA1)	-0.15	0.08	-0.16	0.03	-0.18	0.03	0.00	1.00	-0.02	1.00	-0.02	1.00
E (IA1)	-0.06	0.74	-0.06	0.75	-0.05	1.00	0.00	1.00	0.00	1.00	0.00	1.00
F (IA2)	-0.01	1.00	-0.03	1.00	0.00	1.00	-0.01	1.00	0.01	1.00	0.02	1.00
H (IB)	0.10	0.19	0.02	1.00	0.05	1.00	-0.07	0.50	-0.04	1.00	0.03	1.00
G (IC)	0.00	1.00	0.00	1.00	0.00	1.00	0.00	1.00	0.00	1.00	0.00	1.00
K (II)	0.11	0.23	0.04	1.00	0.15	0.09	-0.06	1.00	0.04	1.00	0.10	0.56
L (III)	0.00	1.00	0.02	1.00	0.00	1.00	0.03	0.99	0.00	1.00	-0.03	1.00
unknown	0.00	1.00	-0.02	0.00	0.00	1.00	-0.02	0.00	0.00	1.00	0.02	0.00

Supplementary Table 3 Antimicrobial activity of staphylococcal strains against *S. aureus* DSM799, *C. acnes* DSM1897 and *C. acnes* 30.2.L1

indicator strain / staphylococcal strain	***S. aureus* DSM 799**	***C. acnes* DSM 1897**	***C. acnes* 30.2.L1**
S. capitis HAB177	-	+	-
S. capitis HAB198	-	+	-
S. capitis HAB200	-	+	-
S. capitis HAB276	-	+	-
S. capitis HAB277	-	+	-
S. capitis HAB278	-	+	-
S. capitis HAB280	-	+	-
S. capitis HAB56	-	+	-
S. capitis HAC49	-	+	-
S. capitis HAC509	-	+	-
S. epidermidis HAA531	+	-	-
S. epidermidis HAA534	+	-	-
S. epidermidis HAB357	+	-	-
S. epidermidis HAB358	+	-	-
S. epidermidis HAB359	+	-	-
S. epidermidis HAB360	+	-	-
S. epidermidis HAB440	+	-	-
S. epidermidis HAC26	+	+	+
S. epidermidis HAC526	+	-	-
S. epidermidis HAC527	+	-	-
S. epidermidis HAC528	+	-	-
S. epidermidis HAC529	+	-	-
S. epidermidis HAC530	+	-	-
S. epidermidis HAC588	-	+	-
S. epidermidis HAC590	-	+	-
S. epidermidis HAF242	-	+	-
S. epidermidis HAF243	+	-	-
S. epidermidis HAF424	-	+	-
S. epidermidis HAF521	+	-	-
S. epidermidis HAF522	+	-	-
S. epidermidis HAF523	+	-	-
S. epidermidis HAF525	+	-	-
S. hominis HAA254	-	+	-
S. hominis HAA272	-	+	-
S. hominis HAA273	-	+	-
S. hominis HAA274	-	+	-
S. hominis HAB257	-	+	-
S. hominis HAC286	+	+	-
S. warneri HAA271	+	-	-
S. warneri HAA333	+	+	-
S. warneri HAA334	+	+	-

Supplementary Table 4 GenBank accession numbers and origin of indicator strains

Indicator strains	SLST-type	Accession number	origin
S. aureus DSM799	-	JXHV00000000	Germany, DSMZ
C. acnes DSM1897	A1	AWZZ00000000	Germany, DSMZ
C. acnes 12.1.L1	A1	CP012354	Scholz et al. 2016 [1]
C. acnes 15.1.R1	C1	CP012355 (chromosome); CP012356 (plasmid)	Scholz et al. 2016 [1]
C. acnes 30.2.L1	D1	CP012350	Scholz et al. 2016 [1]
C. acnes 09-193	D1	LKVE01000000	Davidsson et al. 2017[2]
C. acnes 11-90	H1	MVCG00000000	Davidsson et al. 2017[2]
C. acnes KPA171202	H2	AE017283	Brüggemann et al. 2004[3]
C. acnes 21.1.L1	H1	CP012351	Scholz et al. 2016 [1]
C. acnes 11-49	K1	MVCN00000000	Davidsson et al. 2017[2]
C. acnes 11-79	K2	MVCO00000000	Davidsson et al. 2017[2]
C. acnes PMH5	L1	LJAS00000000	Petersen et al. 2015[4]

Supplementary references:

1. Scholz CF, Brüggemann H, Lomholt HB, Tettelin H, Kilian M. Genome stability of *Propionibacterium acnes*: a comprehensive study of indels and homopolymeric tracts. *Sci Rep* **6**, 20662 (2016).

2. Davidsson S*, et al.* Prevalence of Flp Pili-Encoding Plasmids in *Cutibacterium acnes* Isolates Obtained from Prostatic Tissue. *Front Microbiol* **8**, 2241 (2017).

3. Brüggemann H*, et al.* The complete genome sequence of *Propionibacterium acnes*, a commensal of human skin. *Science* **305**, 671-673 (2004).

4. Petersen R, Lomholt HB, Scholz CF, Brüggemann H. Draft Genome Sequences of Two *Propionibacterium acnes* Strains Isolated from Progressive Macular Hypomelanosis Lesions of Human Skin. *Genome Announc* **3**, (2015).
