# Supplementary material for: Interference and co-existence of staphylococci and Cutibacterium acnes within the healthy human skin microbiome
Source: Commun Biol. 2022 Sep 7;5:923. doi: 10.1038/s42003-022-03897-6 (PMC9452508; doi:10.1038/s42003-022-03897-6)
Supplement: Supplementary file 3 — Description of Additional Supplementary Files [file 42003_2022_3897_MOESM3_ESM.pdf]

Description of Additional Supplementary Files

File name: Supplementary Data 1
Description: Relative abundances (in %) of staphylococci in 93 samples.

File name: Supplementary Data 2
Description: Accession numbers and sequence types of 69 S. epidermidis genomes sequenced in this study.

File name: Supplementary Data 3
Description: Relative abundances (in %) of C. acnes SLST types in 113 samples.

File name: Supplementary Data 4
Description: Differentially expressed genes of S. epidermidis HAF242 (SE): growth of SE in coculture with C. acnes 30.2.L1 (D-class) versus growth of SE in co-culture with C. acnes DSM1897 (A-class).

File name: Supplementary Data 5
Description: Differentially expressed genes of S. epidermidis HAF242 (SE): growth of SE in coculture with C. acnes DSM1897 (A-class) versus growth of SE in monoculture.

File name: Supplementary Data 6
Description: Differentially expressed genes of S. epidermidis HAF242 (SE): growth of SE in coculture with C. acnes 30.2.L1 (D-class) versus growth of SE in monoculture.

File name: Supplementary Data 7
Description: Origin of bacterial isolates (n=572) obtained in this study.

File name: Supplementary Data 8
Description: Database accession numbers of 286 S. epidermidis genomes taken for phylogenetic analyses of this study.

File name: Supplementary Data 9
Description: Accession numbers and SLST type assignments of 75 C. acnes genomes included in the pan-genome analysis of this study.

File name: Supplementary Data 10
Description: raw data for Fig. 1c, 2c, 4c, 5c, S4.
