# Supplementary material for: Interference and co-existence of staphylococci and Cutibacterium acnes within the healthy human skin microbiome
Source: Commun Biol. 2022 Sep 7;5:923. doi: 10.1038/s42003-022-03897-6 (PMC9452508; doi:10.1038/s42003-022-03897-6)
Supplement: Supplementary file 14 — reporting summary [file 42003_2022_3897_MOESM14_ESM.pdf]

nature research

Corresponding author(s): Holger Brüggemann
Charlotte Marie Ahle

Last updated by author(s): Aug 4, 2022

Reporting Summary

Nature Research wishes to improve the reproducibility of the work that we publish. This form provides structure for consistency and transparency in reporting. For further information on Nature Research policies, see our Editorial Policies and the Editorial Policy Checklist.

Statistics

For all statistical analyses, confirm that the following items are present in the figure legend, table legend, main text, or Methods section.

n/a	Confirmed	
☐	☒	The exact sample size (*n*) for each experimental group/condition, given as a discrete number and unit of measurement
☐	☒	A statement on whether measurements were taken from distinct samples or whether the same sample was measured repeatedly
☐	☒	The statistical test(s) used AND whether they are one- or two-sided<br>*Only common tests should be described solely by name; describe more complex techniques in the Methods section.*
☒	☐	A description of all covariates tested
☐	☒	A description of any assumptions or corrections, such as tests of normality and adjustment for multiple comparisons
☐	☒	A full description of the statistical parameters including central tendency (e.g. means) or other basic estimates (e.g. regression coefficient) AND variation (e.g. standard deviation) or associated estimates of uncertainty (e.g. confidence intervals)
☐	☒	For null hypothesis testing, the test statistic (e.g. *F*, *t*, *r*) with confidence intervals, effect sizes, degrees of freedom and *P* value noted<br>*Give P values as exact values whenever suitable.*
☒	☐	For Bayesian analysis, information on the choice of priors and Markov chain Monte Carlo settings
☒	☐	For hierarchical and complex designs, identification of the appropriate level for tests and full reporting of outcomes
☒	☐	Estimates of effect sizes (e.g. Cohen's *d*, Pearson's *r*), indicating how they were calculated

Our web collection on statistics for biologists contains articles on many of the points above.

Software and code

Policy information about availability of computer code

Data collection	No software was used for data collection.
Data analysis	Data analysis was performed with the following software: QIIME2 (v. 2019.7), R (v. 4.0.1 and 4.0.3), Parsnp (v 1.0), CGE Mlst Tool (v 1.0), iTOL (v 5.7), anvi'o (v 7.1), Salmon (v 1.5.2).<br>The following R packages were used: ggplot2 (v. 3.3.5), phyloseq (v 1.34.0), gplots (v 3.1.1.), corrplot (v 0.90), ANCOMBC (v 1.0.5), pheatmap (v 1.0.12), EnhancedVolcano (v 1.8.0), ANCOMBC (v 1.6.0), tximport (v 1.18.0), DeSeq2 (v 1.30.0), apeglm (v 1.12.0).

For manuscripts utilizing custom algorithms or software that are central to the research but not yet described in published literature, software must be made available to editors and reviewers. We strongly encourage code deposition in a community repository (e.g. GitHub). See the Nature Research guidelines for submitting code & software for further information.

Data

Policy information about availability of data

All manuscripts must include a data availability statement. This statement should provide the following information, where applicable:

- Accession codes, unique identifiers, or web links for publicly available datasets
- A list of figures that have associated raw data
- A description of any restrictions on data availability

Whole genome sequence data (69 S. epidermidis genomes) generated for this study is deposited in GenBank with the bioproject number PRJNA793831 and can be accessed here: https://www.ncbi.nlm.nih.gov/bioproject/PRJNA793831. The closed whole genome sequence of S. epidermidis HAF242 is deposited in GenBank with the accession numbers CP090941 (chromosome) and CP090942-CP090944 (plasmids). The amplicon-based NGS data is stored at SRA with the bioproject number PRJNA795320 and can be accessed here: https://www.ncbi.nlm.nih.gov/bioproject/PRJNA795320.
The transcriptome sequencing data (S. epidermidis HAF242 in mono- and co-cultures) is stored at SRA with the bioproject number PRJNA801462 and can be

accessed here:
https://www.ncbi.nlm.nih.gov/bioproject/PRJNA801462.

Field-specific reporting

Please select the one below that is the best fit for your research. If you are not sure, read the appropriate sections before making your selection.

☒ Life sciences ☐ Behavioural & social sciences ☐ Ecological, evolutionary & environmental sciences

For a reference copy of the document with all sections, see nature.com/documents/nr-reporting-summary-flat.pdf

Life sciences study design

All studies must disclose on these points even when the disclosure is negative.

Sample size	Appropriate sample size was chosen based on availability of healthy volunteers and based on experience from previous studies.
Data exclusions	No data were excluded from the study/analyses.
Replication	Experimental validation was achieved by at least three replications.
Randomization	Randomization was not relevant in this study (no grouping was performed).
Blinding	Blinding was not relevant in this study (no grouping was performed).

Reporting for specific materials, systems and methods

We require information from authors about some types of materials, experimental systems and methods used in many studies. Here, indicate whether each material, system or method listed is relevant to your study. If you are not sure if a list item applies to your research, read the appropriate section before selecting a response.

Materials & experimental systems

n/a	Involved in the study	
☒	☐	Antibodies
☒	☐	Eukaryotic cell lines
☒	☐	Palaeontology and archaeology
☒	☐	Animals and other organisms
☐	☒	Human research participants
☒	☐	Clinical data
☒	☐	Dual use research of concern

Methods

n/a	Involved in the study	
☒	☐	ChIP-seq
☒	☐	Flow cytometry
☒	☐	MRI-based neuroimaging

Human research participants

Policy information about studies involving human research participants

Population characteristics	30 healthy human volunteers (female, n=14; male, n=16) with an age range of 22-43 years.
Recruitment	Study participants were recruited from the Beiersdorf volunteer pool. All volunteers were medically assessed prior to inclusion in the volunteer pool. All included volunteers had no history of skin diseases and no topical skin treatment or antibiotics were administered in the last six months. Volunteers refrained from applying any skin cosmetic products on the day of the study. Volunteers received information about the nature and scope of the study (verbal and in writing).
Ethics oversight	Written informed consent was obtained from all volunteers and the study was approved by International Medical & Dental Ethics Commission GmbH (IMDEC), Freiburg (Study no. 67885). Study design complied with all relevant ethics regulations, alining with the Declaration of Helsinki.

Note that full information on the approval of the study protocol must also be provided in the manuscript.
